# Krankheitsmodifizierende Therapie mit Lecanemab bei früher Alzheimer-Demenz

**DOI:** 10.1055/a-2681-4558

**Published:** 2025-10-06

**Authors:** Niels Hansen, Jens Wiltfang

**Affiliations:** 1 27177 Klinik für Psychiatrie und Psychotherapie, Universitätsmedizin Göttingen, Göttingen, Germany; 2 Deutsches Zentrum für Neurodegenerative Erkrankungen (DZNE), Göttingen, Germany; 3 Biomedizinisches Institut (iBiMED), Universität von Aveiro, Aveiro, Portugal

**Keywords:** Alzheimer-Krankheit, Lecanemab, Krankheitsmodifizierende Therapie, Alzheimer's disease, Lecanemab, Disease-modifying therapy

## Abstract

Die Alzheimer Krankheit (AD) ist eine schwere und fortschreitende neurodegenerative Erkrankung des Gehirns, die bislang mit primär nur symptomatisch wirksamen medikamentösen und nicht-medikamentösen Therapieformen als Standardtherapie behandelt wird. Nach der Zulassung des monoklonalen Anti-Amyloid Antikörper durch die FDA (Food and Drug Administration) hat sich die AD-Therapie gewandelt, da es durch diese Therapie möglich geworden ist, den biologischen Krankheitsprozess der AD zu verlangsamen. Lecanemab ist durch den Ausschuss für Humanarzneimittel (CHMP) der Europäischen Arzneimittel-Agentur (EMA) für die Zulassung bei Patienten mit früher AD unter zwei Bedingungen empfohlen worden. Erstens sollen homozygote ApoE4 Träger und zweitens Patienten, die eine orale Antikoagulantien erhalten, Lecanemab nicht erhalten. In der folgenden narrativen Übersicht werden der Wirkmechanismus, die Sicherheit sowie die Nebenwirkungen von Lecanemab erläutert. Ferner werden Risikofaktoren für Nebenwirkungen beschrieben. Schließlich werden die ersten Erfahrungen mit Lecanemab aus den Vereinigten Staaten berichtet sowie die Wirksamkeit und finanzielle Aspekte diskutiert. Lecanemab führt zu einer vorübergehenden Reduktion der Amyloid-ß Ablagerungen und zu einem Benefit, der sich in der Alltagskompetenz, Kognition und Lebensqualität abbilden lässt und durch seine nachweisbare biologisch-klinische nachweisbare Wirksamkeit als ein Durchbruch der AD-Therapie bezeichnet werden kann.

## Abstract (en)

Alzheimer’s disease (AD) is a severe and progressive neurodegenerative disease of the brain that has so far been treated with symptomatic drug and non-drug therapies as standard treatment. Following the approval of the monoclonal anti-amyloid antibody by the FDA, AD therapy has changed, as this therapy has made it possible to attenuate the biological disease process of AD. Lecanemab has been recommended by the Committee for Medicinal Products for Human Use (CHMP) of the European Medicines Agency (EMA) for approval in patients with early AD under two conditions. Firstly, homozygous ApoE4 carriers and secondly, patients receiving oral anticoagulants should not receive lecanemab. The following narrative review explains the mechanism of action, safety and side effects of lecanemab. Furthermore, risk factors for side effects are described. Finally, the first experiences with lecanemab are reported and the efficacy and financial aspects are discussed. Lecanemab leads to a temporary reduction in amyloid-ß deposits and to a benefit that can be reflected in everyday competence, cognition and quality of life and can be described as a breakthrough in AD’s therapy due to its demonstrable biological and clinical efficacy.

## Alzheimer-Demenz und seine Vorstufen

Die Alzheimer-Krankheit (AD) als Ursache der Alzheimer-Demenz ist eine mit erheblichen Einschränkungen einhergehende Erkrankung des Gehirns, die in ihrer Häufigkeit aufgrund der steigenden Lebenserwartung deutlich anwachsen wird. 2060 werden weltweit wahrscheinlich 13,8 Millionen Menschen erkrankt sein 1 . Die Folgekosten der Alzheimer-Demenz sind sowohl für die Gesellschaft als auch die medizinische Versorgung immens. Es wird davon ausgegangen, dass die globalen Gesundheitskosten für Patient*innen mit Demenz 1313,4 Milliarden US Dollar für 55,2 Millionen von Menschen mit Demenz betragen 2 . Modellberechnungen haben ergeben, dass der Tod aufgrund einer Alzheimer-Demenz im Jahre 2040 an zweiter Stelle weltweit nach dem Tod der ischämischen Herzkrankheit rangiert 3 . Zudem ist interessant, dass die globalen Studien zur Krankheitslast zeigen, dass es im Zeitraum von 1990 bis 2019 einen Anstieg der Inzidenz der Alzheimer-Demenz von 507, 96 auf 569,39 pro 100 000 Menschen zu verzeichnen gab 4 . Diese Zahlen verdeutlichen die Notwendigkeit, die Behandlung der AD voranzubringen. Amyloid-β Ablagerungen im Gehirn nehmen im Rahmen des Fortschreitens der AD zu und sind weiterhin neben der Tau-Pathologie 5 und einer Beteilung des Immunsystems mit einer Neuroinflammation wesentlicher Bestandteil der Alzheimer Pathologie. Gerade in frühen Stadien der AD wie bei den Demenzvorstufen der subjektiv kognitiven Beeinträchtigung (SCD) ohne testpsychologisch verifizierbare kognitive Defizite oder einer leichten kognitiven Beeinträchtigung (MCI) ohne wesentliche Beeinträchtigung der Alltagskompetenz ist die Amyloid-Pathologie bereits über Biomarker im Liquor (erniedrigte Ratio Aβ42/40) nachweisbar, die bereits vor einer nachweisbaren Tau Pathologie (erhöhtes p-tau217) auftritt 6 . Somit ist der Nachweis einer Amyloid-Pathologie im Liquor und bald auch im Blutplasma oft die erste Spur einer Alzheimer-Krankheit 6 . Es sollte jedoch hervorgehoben werden, dass der Marker p-tau217 mit der Amyloid-Pathophysiologie zusammenhängt und in den S-3 Demenz Leitlinien 7 derzeit weiterhin p-tau181 im Liquor als der relevante Tau Marker für eine AD neben der Ratio Aβ42/40 im Liquor gilt. Das Gesamt Tau Protein im Liquor dient zur Erfassung der Ausprägung des neurodegenerativen Abbaus bei der AD.

Bisherige pharmakologische Optionen haben nicht die Entfernung der Amyloid-β Ablagerungen zum Ziel, sondern basieren auf einer Neurotransmittermodulation. Solche Therapieoptionen greifen beispielsweise in das cholinerge Defizit bei der Alzheimer-Krankheit durch einen Acethylcholinesterasehemmer ein oder verhindern durch einen N-Methyl-D-Aspartat Rezeptor (NMDAR) Antagonismus die durch eine verstärkte glutamaterge synaptische Transmission hervorgerufene Neurotoxizität. Bislang werden nach den S3-Leitlinien für Demenzen 7 nur solche rein symptomatische Therapien bei der AD empfohlen wie die Verabreichung von Acethylcholinesterasehemmern zur Kompensation des cholinergen Defizits oder dem NMDAR-Antagonisten Memantin zur Verhinderung einer Neurotoxizität. Mittlerweile gibt es jedoch auch Medikamente zur Therapie der Alzheimer-Krankheit, die nicht nur auf der symptomatischen Ebene, sondern auch krankheitsmodifizierend wirken. In den USA sind als Vertreter dieser Sparte bereits drei verschiedene Anti-Amyloid Antikörper wie Aducanumab, Donanemab und Lecanemab von der FDA (Food and Drug Administration) zugelassen worden. Aducanumab wird inzwischen in den USA nicht mehr vermarktet, da die in der Zulassung geforderte zusätzliche Studie zur klinischen Effektivität des Aducanumab vorzeitig 2024 abgebrochen worden ist. Aufgrund der CLARITY-AD Studie 8 ist Lecanemab neben den USA auch in anderen Ländern zugelassen worden wie in China, Hong- Kong, Israel, Südkorea und den Vereinigten Arabischen Emiraten. In Europa ist am 14. November 2024 durch den Ausschuss für Humanarzneimittel (CHMP) der Europäischen Arzneimittel-Agentur (EMA) nach einer erneuten Prüfung der Daten, die auf Antrag des Herstellers Eisai durchgeführt wurde, die Zulassung von Lecanemab für die Behandlung von Patienten im Frühstadium der AD empfohlen worden. Im April 2025 wurde Lecanemab von der Europäischen Kommission für den europäischen Wirtschaftsraum zugelassen.

## Wirkmechanismus von Lecanemab

Lecanemab ist ein monoklonaler humanisierter Anti-Amyloid Antikörper, der sich mit hoher Affinität vorwiegend gegen Amyloid-β Protofibrillen richtet 8 9 , aber auch an Amyloid Oligomere und unlösliche Fibrillen bindet 10 . Die Affinität ist 10–15-mal größer für Protofibrillen als für Monomere 11 . Insbesondere Amyloid-β Protofibrillen sind für Nervenzellen toxischer im Vergleich zu unlöslichen Fibrillen sowie Monomeren 8 12 . Insgesamt ist das bevorzugte Epitop des Immunoglobulin 1 Antikörper Lecanemab jedoch das lösliche aggregierte Amyloid-β 13 . Das Epitop beinhaltet eine kurze Aminosäuren lange Peptidsequenz. Das lösliche Aggregat von dem Amyloid-β Peptid ist die Zielstruktur von Lecanemab. In der Literatur wird als Epitop die Sequenz der Aminosäuren von 1-16 bei dem N-Terminus des Amyloid-β Peptid angegeben 14 . Im Tierexperiment konnte zudem gezeigt werden, dass Lecanemab die Zusammenlagerung aus Fibrinogen und Amyloid-β trennt und auf diese Weise eine durch Fibrinogen vermehrte synaptische Toxizität von Amyloid-β verhindert 15 . In der 18 Monate andauernden Phase 3 Studie bei früher AD (CLARITY AD) konnte gezeigt werden, dass Lecanemab durch die Bindung an Amyloid-β zu einer substanziellen Reduktion der Amyloid-Ablagerungen im Amyloid-PET führt (Verminderung von −59,12 Centiloids gegenüber der Placebogruppe) 8 . Centiloids stellt eine Maßeinheit zur quantitativen Messung der Amyloid-β Ablagerungen im Amyloid-PET dar. Die Veränderungen der Amyloid-Ablagerungen im Amyloid PET wurden bei 698 Patienten in einer Substudie als sekundärer Endpunkt der Studie betrachtet 8 . Darüber hinaus wurden Blutbiomarker der AD im Studienverlauf bestimmt, um diese Amyloid-Rückbildung im Amyloid-PET auf Ebene der Blutbiomarker abzubilden. Tatsächlich konnte gezeigt werden, dass die Ratio Aβ42/40 im Blut angestiegen ist und die Blutplasmaspiegel von p-tau181 und GFAP relativ zu den Placebo-behandelten Patienten deutlich geringer ansteigen 8 . Auch im Liquor konnten Veränderungen der spezifischen Alzheimer-Biomarker beobachtet werden, die für eine Rückbildung der Pathologie sprechen wie ein nach 18 Monaten reduziertes p-tau181 Level und eine ansteigende Ratio Aβ42/40 8 . Somit konnte auf der Ebene der Blutbiomarker gezeigt werden, dass der Einsatz von Lecanemab zu einer biologischen Modifikation der pathophysiologischen Korrelate der Amyloid-β und Tau Pathologie führt.

## Klinische Effektivität von Lecanemab

Insgesamt wurden in der Phase 3 Studie mit Lecanemab bei früher AD 1795 Patientinnen rekrutiert 8 . 898 erhielten den Anti-Amyloid Antikörper Lecanemab und 897 erhielten eine Placebo Behandlung 8 . Der primäre Endpunkt der Studie war die Veränderung der Alltagsfertigkeiten und des Schweregrads der Demenz im Zeitverlauf, die anhand der Demenz Rating Skala (CDR-Sum of Boxes=CDR-SB) eingeschätzt wurde. Die CDR-SB ist ein Instrument, um den Schweregrad der Demenz bei Patienten über sechs kognitive und funktionale Bereiche zu evaluieren wie Gedächtnis, Orientierung, Urteilsvermögen und Problemlösung, gemeinschaftliche Angelegenheiten, Hobbies und persönliche Pflege. Es konnte nach 18 Monaten Behandlung mit Lecanemab eine geringere Verschlechterung der Fähigkeiten im Rahmen der Demenz im Vergleich zu Placebo (der Unterschied betrug 0,45 Punkte im CDR-SB; Konfidenzintervall 95%, −0,67, −0,23, p<0,001) ( Tab. 1 ) erzielt werden 8 . Es sollte jedoch berücksichtigt werden, dass der mittlere Score der Teilnehmer*innen bei Einschluss 3,17 Punkte im CDR-SB für die Verumgruppe und 3,22 für die Placebogruppe betrug. Wichtig ist dies zu beachten, da der Effekt mit einem Weniger an Verschlechterung von 0,45 anders zu gewichten ist, wenn sich dieser auf 3,2 Punkte bezieht und nicht auf den Gesamtbereich von 18 Punkten im CDR-SB. Wenn man 0,45/3,2 betrachtet, ist der Unterschied erheblich größer, als wenn der Bezugspunkt 18 ist (0,45/18). Dies ist insbesondere relevant, da häufig argumentiert wird, dass der klinische Effekt auf den Gesamtbereich der Skala bezogen sehr gering sei. Der Effekt von Lecanemab bei früher AD war in der 18-monatigen Weiterführung in Form der OLE (Open label extension) – Studie ähnlich und anhaltend (Reduktion des CDR-SB um −0,95 Punkte bei Lecanemab gegenüber dem beobachteten kognitiven Abbau in der ADNI [Alzheimer’s Disease Neuroimaging Initiative] Kohorte) 16 . Eine andere Studie ergab ebenso eine geringere, aber nicht signifikante Verschlechterung der Fähigkeiten im Rahmen der Demenz (CDR-SB nach einer Lecanemab-Behandlung im Vergleich zur Placebo-Behandlung von −0,31 (90% Konfidenzintervall −0,620, 0,017; p=0,119) 13 . Zudem konnte auch eine geringere Verschlechterung der kognitiven Fähigkeiten (Reduktion des ADAScog14 [Alzheimer’s Disease Assessment Scale-Cognitive Subscale 14] Scores um −1,44 (95% Konfidenzintervall −2,27; −0,61; p<0,001) als Differenz im Vergleich zu Placebo) bei 854 AD-Patienten mit Lecanemab Therapie gegenüber 872 AD-Patienten mit Placebo-Behandlung beobachtet werden ( Tab. 1 ) 8 . Darüber hinaus ergab sich ebenso in einer anderen Skala zur Beurteilung der kognitiven Fertigkeiten eine geringe Verschlechterung nach Lecanemab bei AD-Patienten im Vergleich zu Placebo (Reduktion des ADCOMS [Alzheimer’s disease Composite Score] Wertes um −0,050 [95% Konfidenzintervall −0,074; −0,027; p<0,001], Tab. 1 ) 8 . Zudem ergab sich eine Steigerung der Alltagsfertigkeiten (Steigerung des ADCS-MCI-ADL [Activities of Daily Living for Mild Cognitive Impairment] Wertes um 2 [95% Konfidenzintervall 1,2; 2,8; p<0,001]) bei 783 AD-Patienten mit Lecanemab Therapie gegenüber 796 AD-Patienten, die eine Placebo Behandlung erhielten ( Tab. 1 ) 8 . In einem Simulations-Modell mit Integration der CLARITY AD Daten konnte gezeigt werden, dass eine mittlere Verzögerung von etwa 2,95 Jahren bei AD-Patienten durch Lecanemab als Zusatztherapie zur Standardtherapie erreicht werden konnte 17 . Das entspricht einer gewonnenen Lebenszeit von in etwa 3 Jahren für AD-Patienten mit Lecanemab Behandlung über einen Lebenszeitraum.

**Table:** **Tab. 1** Klinischer Effekt von Lecanemab.

Klinischer Bereich	Verwendete Skala	Mittlerer Differenz-Effekt gegenüber der Placebo Behandlung	Referenz
Gesundheitsstatus	EQ-5D-5L	49%	[18]
Kognition	ADASCog14	−1,44 (95% KI −2,27; −0,61#)	[8]
Kognition	ADCOMS	−0,050 (−0,074; −0,027#)	[8]
Lebensqualität	ADCS-MCI-ADL	2 (1,2; 2,8#)	[8]
Lebensqualität	QOL-AD	56%	[18]
Lebensqualität	ZBI	38%	[18]
Lebensqualität	QUALYs	0,64	[17]
Schwere der Demenzsymptome	CDR-SB	−0,45 (95% KI −0,67; −0,23#), (−0,95*), −0,31 (90% KI −0,62; 0,02**)	[8] ([16]*), [13]

**Abkürzungen:** ADASCog14=Alzheimer’s Disease Assessment Scale-Cognitive Subscale14, ADCOMS=Alzheimer’s disease Composite Score, ADCS-MCI-ADL=Activities of Daily Living for Mild Cognitive Impairment, CDR-SB=Clinical Dementia Rating – Sum of Boxes, EQ-5D-5L=5-level EQ-5D version, KI=Konfidenzintervall, QOL-AD=Quality of life in Alzheimer’s disease, QUALYs=Quality adjusted life years, ZBI=Zarit Burden Interview. *Ergebnisse der (OLE) Open Label Extension Studie, # p<0,001, **p=0,119

## Lebensqualität nach Therapie mit Lecanemab

Im Rahmen der CLARITY AD Studie wurde zudem die Lebensqualität nach Lecanemab- Behandlung untersucht. Es wurden verschiedene Instrumente zur Messung der Lebensqualität und des Gesundheitsstatus herangezogen wie die EQ-5D-5L (5-level EQ-5D Version) als Patientenbeurteilung für den Gesundheitsstatus und die QOL-AD (Quality of life in Alzheimer’s disease) als Messinstrument zur Patientenbeurteilung und Angehörigenbefragung 18 . Die Messinstrumente wurden verwendet, um die gesundheitsbezogene Lebensqualität (HRQoL) zu bestimmen, die zu Beginn der Messungen und im Folgenden dreimal alle 6 Monate durchgeführt wurde 18 . Diese HRQoL ist deshalb so relevant, da sie sowohl die Perspektive der Patient*innen als auch die der Angehörigen mit deren jeweiligen Wahrnehmungen einbezieht und auf welche Art und Weise diese Wahrnehmungen andererseits die Krankheit an sich verändern können 18 . Es ergab sich eine weniger starke Reduktion der Lebensqualität um ca. 49% (EQ-5D-5L, bezogen auf die adjustierte mittlere Änderung des Skalenwertes gegenüber der Basisbedingung) oder um 56% (QOL-AD, bezogen auf die adjustierte mittlere Änderung des Skalenwertes gegenüber der Basisbedingung) bei Lecanemab nach 18 Monaten verglichen mit der Placebobehandlung ( Tab. 1 ) 18 . Somit war die HRQoL insgesamt gebessert nach Lecanemab-Behandlung im Vergleich zur Placebo-Behandlung. Zudem wurden die Angehörigen befragt mittels des Zarit Burden Interview (ZBI), um deren Belastung bei der Betreuung von Angehörigen mit früher AD zu erfassen, dass eine weniger starke Belastung für die Angehörigen von 38% nach Lecanemab Behandlung zeigte 18 . Eine Evidenz basierte Modelluntersuchung mit Berücksichtigung der Literatur und der CLARITY AD Studie ergab, dass die qualitätskorrigierten Lebensjahre (QALYs) um 0,64 unter Berücksichtigung der Patienten- und Angehörigenperspektive angestiegen sind 17 . Somit konnte mehrfach gezeigt werden, dass sich die Lebensqualität von sowohl den Demenzerkrankten als auch den Angehörigen durch eine Lecanemab Gabe verbessert hat.

## Sicherheit und Nebenwirkungen von Lecanemab

Lecanemab ist als sicher einzuschätzen, denn die Mortalität betrug 0,7% in der Gruppe, die Lecanemab verabreicht bekamen und 0,8% in der Gruppe, die eine Placebobehandlung erhielten ( Tab. 2 ) 8 19 . In der Gruppe der Lecanemab Behandlung wiesen 14% schwere relevante Nebenwirkungen auf und in 11,3% der Placebo-Behandlung traten solche Nebenwirkungen auf ( Tab. 2 ) 8 19 . Die häufigsten schweren Ereignisse, die zu einem Therapieabbruch von Lecanemab führten, traten in 6,9% in der Gruppe der Patienten mit Lecanemab-Behandlung und bei 2,9% bei Placebo-Behandlung auf 19 . Schwere Ereignisse waren Infusionsreaktionen, Amyloid Bildgebungsanomalien mit Ödemen (ARIA-E), Synkopen und Angina Pectoris 8 . Die häufigsten unerwünschten Ereignisse waren infusionsbezogene Nebenwirkungen bei 26,4% der AD-Patienten mit Lecanemab-Behandlung und bei 7,4% der AD-Patienten, die eine Placebo-Behandlung bekamen 8 . Ernsthafte schwere Ereignisse traten bei 1,2% der mit Lecanemab behandelten, jedoch nicht bei Placebo-behandelten AD-Patienten auf 19 . Darüber hinaus traten ARIA-H mit Mikro- und Makrohämorrhagien auf 8 19 . Eine superfizielle Siderose wurde bei 5,6% der AD-Patienten, die Lecanemab als Therapie erhielten und in 2,3% der AD-Patienten mit einer Placebo-Behandlung beobachtet 19 . Hingegen traten ARIA-E bei 12,6% der AD-Patienten mit Lecanemab und bei 1,7% der AD-Patienten mit Placebo-Behandlung auf ( Tab. 2 ) 8 19 . Es gibt eine Reihe an spezifischen Nebenwirkungen durch Lecanemab, jedoch sind schwerwiegende Nebenwirkungen sehr selten.

**Table:** **Tab. 2** ARIA Nebenwirkungen bei Lecanemab-Therapie versus Placebo in der CLARITY AD und OLE-Studie.

Art der Nebenwirkung	Zielgruppe	CLARITY AD	CLARITY AD	Refe-renzen	OLE	Refe-renzen
		*Lecanemab (n=898)*	*Placebo (n=897)*		*Lecanemab (n=1612)*	
**ARIA-E**	***Insgesamt***	12,6%	1,7%	[8, 19]	13,6%	[19]
	***ApoE4 homozygot***	32,6%	3,8%	[8, 19]	34,5%	[19]
	***ApoE4 heterozygot***	10,9%	1,9%	[8, 19]	11,6%	[19]
	***Nicht ApoE4 Träger***	5,4%	0,3%	[8, 19]	6,5%	[19]
**ARIA-H**	***Insgesamt***	16,9%, 17,3%	8,9%, 9%	[8, 19]	18,5%	[19]
	***ApoE4 homozygot***	38,3%, 39%	21,1%,	[8, 19]	39,8%	[19]
	***ApoE4 heterozygot***	13,8%	8,6%	[19]	16,1%	[19]
	***Nicht ApoE4 Träger***	11,5%	3,8%	[19]	11,9%	[19]
**Mortalität**	***Insgesamt***	0,7%	0,8%	[19]	1,0%	[19]
**Schwerwiegendes unerwünschtes Ereignis**	***Insgesamt***	14%	11,3%	[19]	15%	[19]
**Schwerwiegendes unerwünschtes Ereignis mit ARIA-E**	***Insgesamt***	0,8%	0%	[19]	1,1%	[19]
**Schwerwiegendes unerwünschtes Ereignis mit ARIA-H**	***Insgesamt***	0,2%	0%	[19]	0,6%	[19]

**Abkürzungen** : ARIA-E=Amyloid assoziierte Bildgebungsanomalien mit Ödem, ARIA-H=Amyloid assoziierte Bildgebungsanomalien mit Blutung, OLE=open label extension study

## ApoE4 Genotyp als Risikofaktor für Nebenwirkungen von Lecanemab

Es gibt Hinweise, dass der ApoE4 Genotyp der wichtigste genetische Risikofaktor für die Entwicklung einer sporadischen AD ist 20 und zudem bedeutend ist für die vaskuläre Instabilität, die Patienten zur Mikrohämorrhagien prädisponieren kann 21 . ApoE beeinflusst die Integrität der neurovaskulären Funktion, kann die Immunregulation beeinträchtigen und kann zudem zur Akkumulation von Amyloid-β bei der cerebralen Amyloid Angiopathie (CAA) führen 22 . ApoE weist in Menschen drei verschiedene Allele auf: ApoE2, ApoE3 und ApoE4. Während die ApoE2 das Risiko für eine AD reduziert, steigert das ApoE4 Allel das Risiko für eine cerebrale Amyloid Angiopathie und andere Kopathologien der AD wie cerebrovaskuläre Erkrankungen 23 . Die ApoE4 Allelfrequenz beträgt ca. 14% bei der Allgemeinbevölkerung und bis zu 37% bei der AD 20 . In der CLARITY AD Studie waren 53% heterozygot und 16% homozygot bezüglich der Ausprägung des ApoE4 Allels 8 . ApoE4 Träger haben ein gesteigertes Risiko für die Ausbildung von ARIA, die entweder symptomatisch oder wiederholt aufgetreten sind 19 . Zudem haben ApoE4 Träger auch ein erhöhtes Risiko für die cerebrale Amyloidangiopathie mit Inflammation (CAA)-ri / Amyloid-β bezogene Angiitis (ABRA), die zudem ein Risikofaktor für die Entwicklung einer ARIA darstellt 10 . In der CLARITY AD Studie wurden ARIA-E bei heterozygoten ApoE4 Trägern bei 10,9% und in homozygoten ApoE4 Trägern bei 32,6% und bei ApoE4 Nicht Trägern bei 5,4% beobachtet ( Tab. 2 ) 8 . Symptomatische ARIA-E ereigneten sich bei heterozygoten ApoE4 Trägern mit AD bei 1,7%, bei homozygoten ApoE4 Trägern mit AD bei 9,2% und bei nicht ApoE4 Trägern mit AD wurden diese nur bei 1,4% beschrieben 19 . Insgesamt sind die ARIA-H und ARIA-E bei homozygoten ApoE4 Trägern bei AD-Patienten deutlich häufiger als bei heterozygoten oder Nicht ApoE4 Trägern.

## Vaskuläre Hirnschädigung und Lecanemab

In einer Übersicht 19 wurden die Sicherheitsdaten der CLARITY AD Studie und der OLE -Studie in Teilnehmern mit früher AD präsentiert. Es wurden bei diesen Sicherheitsdaten die Daten von 1795 Teilnehmern der CLARITY-AD und bei 1612 Teilnehmern mit mindestens einer Dosierung von Lecanemab (CLARITY AD und OLE) ausgewertet. Es gab keine Todesfälle, die auf Lecanemab zurückgeführt werden konnten in der CLARITY AD Studie, jedoch gab es 9 Tote während der OLE-Studie 19 . 4 von diesen 9 Toten stehen mit der Lecanemab Medikation in einem Zusammenhang 19 . Drei dieser Toten konnten auf eine intrazerebrale Blutung zurückgeführt werden 19 . Es zeigten sich ARIA-H bei 8,9% (n=897) der AD-Patienten, die eine Placebo erhielten und bei 16,9% (n=898) der AD-Patienten, die Lecanemab einnahmen in der CLARITY AD Studie, während in der CLARITY AD und OLE-Studie diese bei 18,5% (n=1612) auftraten ( Tab. 2 ) 19 . Intrazerebrale Blutungen (CLARITY AD: Placebo 0,1%, Lecanemab 0,6%; OLE: Lecanemab 0,5%) waren deutlich weniger häufig bei den AD-Patienten mit Lecanemab als Mikrohämorraghien (CLARITY AD: Placebo: 7,6%, Lecanemab: 14,0%; OLE: Lecanemab 16%) und superfizielle Siderosen (CLARITY AD: Placebo 2,3%, Lecanemab: 5,6%; OLE Lecanemab: 6%) 19 . Für die Risikostratifizierung von AD-Patienten für die Lecanemab Gabe ist es daher erforderlich, die Präsenz von CAA-Biomarkern und die kortikale superfizielle Siderose zu berücksichtigen. Wenn die ApoE Trägerschaft in der CLARITY AD und der OLE-Studie berücksichtigt wird bei der Ausbildung von ARIA-H, ist festzustellen, dass ARIA-H deutlich häufiger bei ApoE4 Homozygoten AD-Patienten (38,3% bei Lecanemab vs. 21,1% bei Placebo bei CLARITY AD, OLE: 39,8% bei Lecanemab) auftraten ( Tab. 2 ) 19 . Diese sind häufiger als bei heterozygoten ApoE4 AD-Patienten (13,8% bei Lecanemab vs. 8,6% vs. Placebo bei CLARITY AD, OLE: 16,1% bei Lecanemab) und deutlich häufiger als bei Nicht ApoE4-Trägern bei AD-Patienten (11,5% bei Lecanemab vs. 3,8% bei Placebo bei CLARITY AD, OLE: 11,9% bei Lecanemab) 19 . Somit wird ersichtlich, dass die homozygote ApoE4 Trägerschaft bei AD-Patienten häufiger mit ARIA-H assoziiert ist und damit nachvollziehbar ein Ausschlussgrund für die Vergabe des Lecanemab sein sollte. Zudem ist es wahrscheinlich, dass ApoE4 mit einem vermehrten Auftreten einer kongophilen Amyloid Angiopathie vergesellschaftet ist 24 .

## Empfehlungen für den Gebrauch des Lecanemab

Alle zwei Wochen wird Lecanemab über eine intravenöse Infusion à 10mg/kg über eine Zeitdauer von einer Stunde appliziert 8 9 10 . Im Mittel hat Lecanemab eine Halbwertszeit von ca. 5,3 Tagen 25 . Eine proteolytische Spaltung führt dazu, dass Lecanemab eliminiert wird 25 . Zudem wird Lecanemab nicht hepatisch metabolisiert oder renal ausgeschieden 25 . Es sollte bei der ersten Gabe für drei weitere Stunden nach der Infusion eine Überwachung stattfinden, um infusionsbezogene Nebenwirkungen zu überwachen 10 . Hingegen kann dies bereits bei der zweiten und dritten Gabe auf 2 Stunden und bei der vierten Gabe auf eine halbe Stunde verringert werden, wenn bis dato sich keine Infusionsreaktionen ereignet haben 10 . Zudem sollte für die frühzeitige Identifikation von ARIA’s eine Magnetresonanztomographie (MRT) nach der 5ten, 7ten und 14ten Infusion ergänzt werden 8 . Darüber hinaus sollte auch ein MRT vor der 26. Infusion insbesondere bei den AD-Patienten, die ApoE4 Träger sind durchgeführt werden 10 . Es ist wichtig, dass Neuroradiologen in der Sichtung der ARIAs geschult werden. Des Weiteren ist es relevant, dass diese auch in Verbindung mit den jeweiligen Klinikern die Erkennung und das Management der Symptome und radiologischen Veränderungen besprochen wird. Es ist beispielsweise für radiologisch nur leichte, aber asymptomatische ARIA sinnvoll, die Lecanemab Infusionen und eine monatliche MRT- Kontrolle durchzuführen 10 . Diese Kontrollen sollten so lange erfolgen, bis ARIA-H sowie ARIA-E stabilisiert haben 11 . Die Infusionen von Lecanemab sollten jedoch nicht fortgeführt werden, wenn hingegen mittelschwer oder schwer ausgeprägte ARIA sich ereignen 11 . Zudem sollten auch AD-Patienten ein monatliches MRT erhalten, bis ARIA-E sich ganz zurückgebildet und die ARIA-H sich in der Ausdehnung stabilisiert haben 11 . Darüber hinaus ist es wichtig, schwere Fälle von ARIA zu erkennen, die einem CAA-ri Syndrom ähnlich sind und ein großflächiges ödematöses Hirnareal einschließen 26 . ARIAs können reversibel sein, aber auch mit dem Tod enden 19 . Nach dem Ausschluss einer cerebralen Ischämie mit Diffusions-gewichteten Sequenzen im MRT, sollte überdacht werden, ob eine hochdosierte Glucocorticoidtherapie à 1 g über 5 Tage mit einer langsamen Reduktion über mehrere Wochen durchgeführt werden sollte 10 . Eine schwer ausgeprägte ARIA, die sich entweder nur in der Bildgebung und/ oder symptomatisch als schwer einstufen lässt, sollte dazu führen, die Lecanemab-Infusionen zu beenden 10 . Aber auch jede großflächige Hirnblutung, mehr als zehn Mikrohämorrhagien oder zwei ARIA-Episoden seit Behandlungsbeginn sollte ebenso einen Grund darstellen, um eine Lecanemab-Behandlung abzubrechen 10 . Aber auch für schwere Infusionsreaktionen sollte die Infusion gestoppt werden und Diphenhydramin (25–50 mg) oder Acetominophen (650–1000 mg) verabreicht werden 10 . Diese Medikamente sollte alle 4–6 Stunden erneut verabreicht werden, bis sich die Symptome komplett zurückgebildet haben. Falls diese Medikamente ineffektiv sind, kann Dexamethason in einer Dosierung von 10–15 mg über 6 Stunden vor der Infusion oder Methylprednisolon in einer Dosierung von 80 mg ebenso 6 Stunden vor der Infusion verabreicht werden 10 .

## Bisherige Erfahrungen mit Lecanemab

Es wurde kürzlich eine Studie publiziert 9 , die zu den ersten Erfahrungen mit der Lecanemab Behandlung bei AD-Patienten in Louisville Norton in den USA berichtet. Es wurden 71 Patienten mit im Liquor bestätigter AD eingeschlossen und es wurde mindestens 1 Gabe von Lecanemab verabreicht 9 . In dieser AD-Patientenpopulation waren 13% homozygote und 51% heterozygote ApoE4 Träger. 24% hatten davon ARIAs 9 . Von den 12 AD-Patienten die ARIAs entwickelt haben waren 4 homozygot und 6 heterozygote ApoE4 Träger 9 . Von den 9 Patienten, die homozygote ApoE4 Träger waren, hatten 44% ARIAs. Von den 36 heterozygoten ApoE4 Träger mit AD hatten 17% ARIAs 9 . Das entspricht einer ähnlichen Dimension und Verteilung der ARIAs bezüglich der ApoE4 Trägerschaft bei den Patienten wie bei den Zulassungsstudien. Jedoch waren die infusionsbezogenen Nebenwirkungen etwas häufiger mit 37% gegenüber 26% bei CLARITY AD 9 . Für die klinische Wirksamkeit liegen noch keine Langzeitdaten vor. Insgesamt kann somit vorübergehend festgehalten werden, dass zwar etwas häufigere infusionsbezogene Nebenwirkungen, aber eine ähnliche Häufigkeit der ARIA vorlagen.

## Diskussion

Lecanemab ist ein Durchbruch in der Therapie der Alzheimer-Krankheit, da diese Substanz auf Biomarker Ebene nachweislich krankheitsmodifizierend ist. Zudem kann auch klinisch eine Verbesserung gegenüber der Placebo-Behandlung um 27% nach den Daten der CLARITY AD Studie demonstriert werden. Noch bedeutender ist wahrscheinlich, dass durch die Lecanemab- Behandlung das Progressionsrisiko innerhalb von Demenzstadien um 31% gesenkt werden kann 27 .

### Kritische Würdigung des klinischen Effekts von Lecanemab

Dennoch gibt eine erhebliche Kontroverse um den klinischen Benefit und die biologische Wirksamkeit von Lecanemab. Eine Gruppe von Autoren geht nach Berechnungen davon aus, dass der Behandlungseffekt mit 2,5% durch Lecanemab wesentlich geringer ist als behauptet 28 . Zudem sei die kognitive Leistungsfähigkeit im ADASCog14 Score in dem Beobachtungszeitraum von 24 Wochen sogar schlechter als nach der Standardbehandlung mit Donepezil 10 mg/d 28 . Andere Autoren berechneten, dass nach Bezug der klinischen Effektivität nur auf die Änderung des CDR-SB, Lecanemab zu einem numerisch kleineren klinischen Effekt auf der CDR-SB von −0,45 als Donepezil mit −0,53 führte 29 . Jedoch gilt es auch hier zu bedenken, dass die Bezugsgröße entscheidend für die Wertigkeit des klinischen Effekts sein kann, je nach Ausgangswert des CDR-SB bei Einschluss. Andererseits konnte in der OLE-Studie ein anhaltender Effekt von insgesamt −0,95 gegenüber der ADNI-Kohorte beobachtet werden, so dass möglicherweise Lecanemab über einen längeren Zeitraum von 3 Jahren effektiver als Donepezil ist. In anderen Skalen wie der ADASCog zeigte Donepezil gegenüber Lecanemab sogar einen klaren Benefit (−2,37 Verlangsamung bei Donepezil versus −1,44 Verlangsamung bei Lecanemab) 29 , so dass im Langzeitverlauf unter kritischer Würdigung Lecanemab wahrscheinlich effektiver ist, obwohl der klinische Unterschied zu Beginn zwischen den Substanzen nicht erheblich ist. Berücksichtigt wird hierbei jedoch nicht, dass es Subpopulationen bei der CLARITY AD Studie gibt, die eine solche Anzahl mitbedingen wie die ApoE4 Trägerschaft und eine orale Antikoagulation. Durch Ausschluss der homozygoten ApoE4 Träger unter den AD-Patienten kann das Auftreten von leichten bis zu moderaten Nebenwirkungen von 10% auf 2% reduziert werden 27 . Patienten mit einer oralen Antikoagulation sollten von der Gabe von Lecanemab grundsätzlich nach den Empfehlungen der CHMP ausgeschlossen werden.

### Geografische Verfügbarkeit und Risikoabschätzung

Weitere Aspekte sind in der Anwendungsplanung von Lecanemab zu berücksichtigen, wie beispielsweise die geografische Zugänglichkeit und Verfügbarkeit des Präparats. In einer Simulationsstudie wurde dies für eine Region in Japan berechnet 30 . Dies ist von Bedeutung, da die Verabreichung von Lecanemab nur in einzelnen Zentren angeboten werden wird, sodass Überlegungen stattfinden müssen, wie solche Zentren erreicht und welche Patienten in solchen Zentren vorranging behandelt werden sollten. Wenn Lecanemab mit anderen Antikörpertherapien im Bereich der Onkologie, der Neurologie und der Rheumatologie verglichen wird, ist Lecanemab sogar kostengünstig 31 . Die Anzahl der zu behandelnden Patienten, um einen Effekt zu erzielen, ist ebenso vergleichbar wie für die Antikörper Ocrelizumab bei der Multiplen Sklerose und beträgt 14–18 31 . Sowohl das Risiko des Todes durch das Medikament ist gering bei 0,1% 31 und auch das Risiko für schwerwiegende Ereignisse während der Behandlung ist mit 7% 31 eher risikoärmeren Bereich der monoklonalen Antikörper einzuordnen, die in der Onkologie oder Neurologie eingesetzt werden. In den USA sind bereits Gruppen von verschiedenen Disziplinen wie Geriatrie, Psychiatrie, Neurologie, Nuklearmedizin, Neuroradiologie eingerichtet worden, um Patienten für die Lecanemab Behandlung zu identifizieren und gemeinsam zu behandeln ähnlich wie dies in Tumorboards bereits praktiziert wird.

### Ökonomische Aspekte der Behandlung mit Lecanemab

Es kann von einem erheblichen Bedarf an Patienten unter Berücksichtigung der Ausschlusskriterien mit früher AD für Lecanemab ausgegangen werden. Die Behandlungskosten betragen für Lecanemab ca. 26 500 US-Dollar pro Jahr. Zudem sind die Kosten für die MRT und Amyloid-Positronen Emissions Tomographie (PET) Untersuchungen zu veranschlagen, die in einer ersten Schätzung bei einem reduzierten Preis von Lecanemab von 9275 US-Dollar ca. 177 000 US-Dollar und somit 168 202,98 Euro bei einem Patienten mit einem günstigen klinischen Verlauf bei Verhinderung einer Progression von MCI zu einer Demenz über 3 Jahre betragen 32 . Somit ist es wichtig, dass nicht nur die reine Medikamenten Applikation berücksichtigt wird, sondern für ein Szenario der mit Lecanemab Gabe in Verbindung stehenden Kosten auch zudem notwendige apparative Kosten für die Bildgebung eingepreist werden. Trotz der hohen Kosten ist Lecanemab aus gesundheitsökonomischen Gründen sinnvoll. Dies liegt daran, dass Modellberechnungen in einer Studie 17 ergeben haben, dass die Kosten aus US-amerikanischer Sicht für Lecanemab zusätzlich zur Standardbehandlung unter Berücksichtigung des Zugewinns von QALYs (0,64) (QUALYs = Quality-adjusted life years, Qualitätsbereinigtes Lebensjahr) und einem Zugewinn an zusätzlichen Lebensjahren sich ökonomisch rentieren. Ganz wichtig ist auch ein Kostenaspekt, der dadurch entsteht, wenn Lecanemab nicht eingesetzt wird. Es gab nämlich Berechnungen, die zeigten, dass eine Behandlung mit Lecanemab zusätzlich zur Standardbehandlung bei einem Zugewinn an Lebensqualität zu einer Reduktion von Nicht-Behandlungskosten führten, die etwa 8707 US-Dollar betragen 17 . Aus diesen Berechnungen geht hervor, dass durch die Lecanemab Behandlung auch erhebliche Einsparungen an Kosten für das Gesundheitssystem innerhalb der Demenzbehandlung möglich sind.

## Fazit für die Praxis

Der Nutzen durch eine Therapie mit Lecanemab hinsichtlich Kognition, Lebensqualität und Alltagskompetenzen überwiegt gegenüber dem Risiko Schäden durch die Therapie zu erleiden, wenn die Ausschlussgründe für eine Therapie wie ApoE4 Homozygotie und orale Antikoagulation berücksichtigt werden.

Die nationale Versorgungsstruktur von Patienten mit AD wird optimiert werden müssen, um die innovative krankheitsmodifizierende Therapie einer möglichst hohen Zahl an Patienten anbieten zu können, die voraussichtlich besonders nachhaltig profitieren werden.

Die Umgestaltung und Etablierung neuer Versorgungskonzepte, die finanziell abgestimmt sein sollten mit den jeweiligen finanziellen Versorgungsträgern, ist eine große Herausforderung für die nächsten Jahre.

## References

[1] Alzheimer’s Association Alzheimer’s disease facts and figures Alzheimer’s Dementia 2023 4 1598 1695 10.1002/alz.13016 36918389

[2] Wimo A Seeher K Cataldi R The worldwide costs of dementia in 2019 Alzheimers Dement 2023 19 2865 2873 36617519 10.1002/alz.12901 PMC10842637

[3] Wang J T Xu G Ren R Y The impacts of health insurance and resource on the burden of Alzheimer’s disease and related dementias in the world population Alzheimers Dement 2023 19 967 979 35820032 10.1002/alz.12730

[4] Su M Wang T Zou C Cao K Liu F Global, regional, and national burdens of Alzheimer’s disease and other forms of dementia in the elderly population from 1999 to 2019: A trend analysis based on the Global Burden of Disease Study 2019 Ibrain 2024 10 488 499 39691425 10.1002/ibra.12181 PMC11649385

[5] Jack C R Andrews J S Beach T G Revised criteria for diagnosis and staging of Alzheimer’s disease: Alzheimer’s Association Workgroup Alzheimers Dement 2024 20 5143 5169 38934362 10.1002/alz.13859 PMC11350039

[6] Teunissen C E Kolster R Triana-Baltzer G Plasma p-tau immunoassays in clinical research for Alzheimer’s disease Alzheimers Dement 2024 10.1002/alz.14397. PMC11977406 39625101

[7] DGN e. V. & DGPPN e. V. (Hrsg.) S3-Leitlinie Demenzen, Version 4.0, 8.11.2023, verfügbar unterhttps://register.awmf.org/de/leitlinien/detail/038-013Zugriff am 16.12.2024.

[8] van Dyck C H Swanson C J Aisen P Lecanemab in Early Alzheimer’s Disease N Engl J Med 2023 388 9 21 36449413 10.1056/NEJMoa2212948

[9] Shields LB E Hust H Cooley S D Initial Experience with Lecanemab and Lessons Learned in 71 Patients in a Regional Medical Center J Prev Alzheimers Dis 2024 11 1549 1562 39559868 10.14283/jpad.2024.159 PMC11573839

[10] Cummings J Apostolova L Rabinovici G D Lecanemab: Appropriate Use Recommendations J Prev Alzheimers Dis 2023 10 362 377 37357276 10.14283/jpad.2023.30 PMC10313141

[11] Arroyo-Pacheco N Sarmiento-Blanco S Vergara-Cadavid G Monoclonal therapy with lecanemab in the treatment of mild Alzheimer’s disease: A systematic review and meta-analysis Ageing Res Rev 2024 104 102620 39638097 10.1016/j.arr.2024.102620

[12] O’Nuallain B Freir D B Nicoll A J Amyloid beta-protein dimers rapidly form stable synaptotoxic protofibrils J Neurosci 2010 30 14411 14419 20980598 10.1523/JNEUROSCI.3537-10.2010 PMC2987723

[13] Swanson C J Zhang Y Dhadda S A randomized, double-blind, phase 2b proof-of-concept clinical trial in early Alzheimer’s disease with lecanemab, an anti-Aβ protofibril antibody Alzheimers Res Ther 2021 13 80 33865446 10.1186/s13195-021-00813-8 PMC8053280

[14] Plotkin S S Cashman N R Passive immunotherapies targeting Aβ and tau in Alzheimer's disease Neurobiol Dis 2020 144 105010 10.1016/j.nbd.2020.105010 32682954 PMC7365083

[15] Singh P K Simões-Pires E N Chen Z L Lecanemab blocks the effects of the Abeta/fibrinogen complex on blood clots and synapse toxicity in organotypic culture Proc Natl Acad Sci U S A 2024 121 e2314450121 38621133 10.1073/pnas.2314450121 PMC11047064

[16] Eisai. New Clinical Data Demonstrates Three Years of Continuous Treatment with Dual-Acting LEQEMBI ^®^ (lecanemab-irmb) Continues to Significantly Benefit Early Alzheimer’s Disease Patients Presented at The Alzheimer’s Association International Conference (AAIC) 2024. URL https://www.eisai.com/news/2024/pdf/enews.202456.pdf

[17] Tahami Monfared A A Ye W Sardesai A Estimated Societal Value of Lecanemab in Patients with Early Alzheimer’s Disease Using Simulation Modeling Neurol Ther 2023 12 795 814 36929345 10.1007/s40120-023-00460-1 PMC10195963

[18] Cohen S van Dyck C H Gee M Lecanemab Clarity AD: Quality-of-Life Results from a Randomized, Double-Blind Phase 3 Trial in Early Alzheimer’s Disease J Prev Alzheimers Dis 2023 10 771 777 37874099 10.14283/jpad.2023.123

[19] Honig L S Sabbagh M N van Dyck C H Updated safety results from phase 3 lecanemab study in early Alzheimer’s disease Alzheimers Res Ther 2024 16 105 38730496 10.1186/s13195-024-01441-8 PMC11084061

[20] Belloy M E Napolioni V Greicius M D A Quarter Century of APOE and Alzheimer’s Disease: Progress to Date and the Path Forward Neuron 2019 101 820 838 30844401 10.1016/j.neuron.2019.01.056 PMC6407643

[21] Cheng Y Xi G Jin H Thrombin-induced cerebral hemorrhage: role of protease-activated receptor-1 Transl Stroke Res 2014 5 472 475 24323711 10.1007/s12975-013-0288-8 PMC3962522

[22] Foley K E Wilcock D M Three major effects of APOEε4 on Aβ immunotherapy induced ARIA Front Aging Neurosci 2024 16 1.412006E6 10.3389/fnagi.2024.1412006 PMC11096466 38756535

[23] Neu S C Pa J Kukull W Apolipoprotein E Genotype and Sex Risk Factors for Alzheimer Disease: A Meta-analysis JAMA Neurol 2017 74 1178 1189 28846757 10.1001/jamaneurol.2017.2188 PMC5759346

[24] Kane M 2024 In: Pratt VM, Scott SA, Pirmohamed M, Esquivel B, Kattman BL, Malheiro AJ, editors. Lecanemab Therapy and APOE Genotype. Medical Genetics Summaries [Internet] Bethesda (MD) National Center for Biotechnology Information (US) 2012 39141762

[25] Yoon C H Groff C Criss O Lecanemab: A Second in Class Therapy for the Management of Early Alzheimer’s Disease Innov Pharm 2024 15 10.24926/iip.v15i1.5787. PMC11107961 38779110

[26] Antolini L DiFrancesco J C Zedde M Spontaneous ARIA-like Events in Cerebral Amyloid Angiopathy-Related Inflammation: A Multicenter Prospective Longitudinal Cohort Study Neurology 2021 97 e1809 e1822 34531298 10.1212/WNL.0000000000012778 PMC8610623

[27] Jessen F Frölich L Hort J Amyloid-lowering treatment in Alzheimer’s disease Lancet 2024 404 2161 2162 39551061 10.1016/S0140-6736(24)02510-8

[28] Espay A J Kepp K P Herrup K Lecanemab and Donanemab as Therapies for Alzheimer’s Disease: An Illustrated Perspective on the DataeNeuro 2024 11ENEURO.0319-23.2024 10.1523/ENEURO.0319-23.2024 PMC11218032 38951040

[29] Burke J F Kerber K A Langa K M Albin R L Kotagal V Lecanemab: Looking Before We Leap Neurology 2023 101 661 665 37479527 10.1212/WNL.0000000000207505 PMC10585683

[30] Ohashi K Takahashi-Iwata I Jieyu Z Sakushima K Yabe I Ogasawara K Are There Shortages and Regional Disparities in Lecanemab Treatment Facilities? A Cross-Sectional Study Health Serv Insights 2024 1711786329241299312 10.1177/11786329241299312 PMC11574910 39563977

[31] Jicha G A Abner E L Coskun E P Perspectives on the clinical use of anti-amyloid therapy for the treatment of Alzheimer’s disease: Insights from the fields of cancer, rheumatology, and neurology Alzheimers Dement (N Y) 2024 10 e12500 39296920 10.1002/trc2.12500 PMC11409193

[32] Perron J Scramstad C Ko J H Analysis of Costs for Imaging-Assisted Pharmaceutical Intervention in Alzheimer’s Disease with Lecanemab: Snapshot of the First 3 Years J Alzheimers Dis 2023 96 1305 1315 37927263 10.3233/JAD-230633

